# The Prognostic Impact of Paroxysmal Atrial Fibrillation on Disability Severity and Activity of Daily Living After Acute Ischemic Stroke

**DOI:** 10.3390/diagnostics15202637

**Published:** 2025-10-19

**Authors:** Marius Militaru, Daniel-Florin Lighezan, Florina Buleu, Stela Iurciuc, Daian Ionel Popa, Adriana Cojocaru, Tiberiu Buleu, Anda Gabriela Militaru

**Affiliations:** 1 Department of Neuroscience, Neurology II University Clinic, “Victor Babes” University of Medicine and Pharmacy, E. Murgu Square, Nr. 2, 300041 Timisoara, Romania; marius.militaru@umft.ro; 2 Emergency City Clinical Hospital Timisoara, Gheorghe Dima Street Nr. 5, 300254 Timisoara, Romania; dlighezan@umft.ro (D.-F.L.); daian-ionel.popa@umft.ro (D.I.P.); militaru.anda@umft.ro (A.G.M.); 3 Centre of Advanced Research in Cardiology and Hemostasology, “Victor Babes” University of Medicine and Pharmacy, E. Murgu Square, Nr. 2, 300041 Timisoara, Romania; 4 Department of Internal Medicine I, Medical Semiology I University Clinic, “Victor Babes” University of Medicine and Pharmacy, E. Murgu Square, Nr. 2, 300041 Timisoara, Romania; 5 Department of Cardiology, “Victor Babes” University of Medicine and Pharmacy, E. Murgu Square No. 2, 300041 Timisoara, Romania; 6 Emergency County Hospital “Pius Brinzeu”, 325100 Timisoara, Romania; tiberiu.buleu@umft.ro; 7 Doctoral School, Faculty of General Medicine, “Victor Babes” University of Medicine and Pharmacy, E. Murgu Square, Nr. 2, 300041 Timisoara, Romania; 8 Research Center for Medical Communication, “Victor Babes” University of Medicine and Pharmacy, 300041 Timisoara, Romania; 9 Department of Neuroscience, Discipline of Pedopsychiatry “Victor Babes” University of Medicine and Pharmacy, E. Murgu Square, Nr. 2, 300041 Timisoara, Romania; adriana.cojocaru@umft.ro; 10 Clinical Department of Child and Adolescent Psychiatry, Children’s Emergency Hospital “Louis Turcanu”, “Victor Babes” University of Medicine and Pharmacy, 2 Eftimie Murgu Square, 300041 Timisoara, Romania; 11 Faculty of Nursing, “Victor Babes” University of Medicine and Pharmacy, 300041 Timisoara, Romania

**Keywords:** atrial fibrillation, acute ischemic stroke, disability, neurological symptoms, activities of daily living

## Abstract

**Background:** The ongoing discourse surrounding the connection between atrial fibrillation (AF) and stroke continues to be a topic of considerable discussion. Atrial fibrillation (AF) is a well-established risk factor for ischemic stroke, yet the prognostic significance of paroxysmal AF in functional recovery remains uncertain. While persistent AF has consistently been associated with more severe strokes and poorer outcomes, evidence regarding paroxysmal AF is limited and conflicting. This research examines how paroxysmal AF influences the severity of post-stroke disability in individuals experiencing acute ischemic stroke. **Materials and Methods:** A total of 236 patients presenting with acute ischemic stroke and cardiovascular risk factors were evaluated upon admission to the Neurology Department. Of these, 118 patients with paroxysmal AF were assigned to Group A, and 118 patients without AF were assigned to Group B. To determine the severity of disability, clinical, neurological, and imaging assessments were performed utilizing the modified Rankin Scale (mRS), Activities of Daily Living (ADL) score, National Institutes of Health Stroke Scale (NIHSS), and Medical Research Council (MRC) scale. **Results:** Patients in Group A exhibited significantly poorer outcomes in comparison to those in Group B, evidenced by lower ADL scores, elevated NIHSS and MRC scores, and increased levels of disability (*p* < 0.05). Within Group A, a stronger correlation was observed between mRS scores and neurological symptoms, motor deficits, and daily functioning. Logistic regression analysis indicated that among all stroke patients (comprising Groups A and B), the probability of experiencing moderate to severe disability (mRS ≥ 3) escalated by 31.6% for each unit increase in NIHSS and diminished by 64.5% for every unit increase in MRC. In Group A, an increase of one unit in ADL correspondingly lowered the risk of mRS ≥ 3 by 22.7%, in contrast to a reduction of 17.8% in the overall stroke population (Groups A and B combined). Additionally, an enhancement in MRC score led to an 83.5% decrease in the risk of disability within Group A, compared to a 75.8% reduction in Group B. Moreover, in Group A, each unit increment in the HAS-BLED score was associated with a 32.5% rise in the risk of severe disability (OR = 1.325; 95% CI: 1.015–1.729; *p* < 0.05). **Conclusions:** Paroxysmal atrial fibrillation was significantly associated with a higher risk of moderate to severe disability following acute ischemic stroke compared to patients without AF. The severity of post-stroke disability in Group A is closely linked to reduced functional independence (lower ADL), more pronounced neurological impairment (higher NIHSS), greater motor deficits (lower MRC), and increased bleeding risk (higher HAS-BLED score). These findings highlight the importance of early identification and comprehensive monitoring of functional, neurological, and cardiovascular parameters in stroke patients with paroxysmal AF. Tailored rehabilitation strategies aimed at improving motor function, daily living activities, and controlling hemorrhagic risk can play a crucial role in reducing long-term disability and enhancing the reintegration of these patients into family and social life.

## 1. Introduction

Stroke is a leading cause of acquired disability and one of the primary contributors to mortality and long-term dependence worldwide [1]. It is also the most frequent reason for acute neurological hospitalization in high-income countries [2]. Despite advancements in treatment options—such as intravenous thrombolysis and mechanical thrombectomy—a considerable number of stroke survivors continue to face enduring functional limitations and a diminished quality of life [3,4].

Atrial fibrillation (AF), recognized as the most prevalent sustained cardiac arrhythmia, serves as a significant independent risk factor for ischemic stroke [5]. It is estimated that AF is responsible for 15–30% of all ischemic strokes, particularly those classified as cardioembolic events [6]. Strokes associated with AF are generally more severe, presenting a greater risk of disability, recurrence, and mortality in comparison to strokes not linked to AF [7,8].

The paroxysmal form of AF, which is characterized by its spontaneous resolution within seven days, has gained increasing acknowledgment for its clinical significance in the pathophysiology of ischemic stroke [9]. Nevertheless, the extent of its influence on functional outcomes and the severity of disability following a stroke remains a topic of ongoing debate. In the literature, no study has directly analyzed this association. Some reports suggest that persistent atrial fibrillation is linked to higher risks [10,11], whereas others describe more favorable outcomes [12,13]. This inconsistency creates uncertainty about whether paroxysmal atrial fibrillation should be managed with the same intensity in prognostic assessment and rehabilitation planning.

Moreover, although CHA_2_DS_2_-VASc and HAS-BLED scores are validated for thromboembolic and bleeding risk prediction [14] their prognostic utility in forecasting post-stroke disability in patients with paroxysmal AF remains poorly understood. This gap in knowledge represents a critical barrier to tailoring secondary prevention and rehabilitation strategies.

Accordingly, the present study aimed to investigate the prognostic significance of paroxysmal AF in functional recovery after acute ischemic stroke, with a particular focus on the predictive value of clinical characteristics, neurological severity, and functional scales, as well as the potential role of CHA_2_DS_2_-VASc and HAS-BLED scores in disability outcomes.

## 2. Materials and Methods

### 2.1. Study Design and Participants

This retrospective observational study was conducted at the Neurology II Department of the Emergency County Hospital Timisoara and Municipal Emergency Hospital Timisoara, Romania. Data were collected from patients admitted between 1 September 2022 and 31 December 2024, for evaluation of their first-ever acute ischemic stroke.

A total of 236 patients were included, based on neurological assessment and neuroimaging—computed tomography (CT) or magnetic resonance imaging (MRI)—confirming the occurrence of acute ischemic stroke. None of these patients underwent treatment with intravenous thrombolysis or mechanical thrombectomy. Acute Ischemic stroke was defined [15,16] as the acute onset of neurological deficits lasting more than 24 h without evidence of hemorrhage on initial CT scan or with corresponding ischemic lesions on diffusion-weighted MRI.

To minimize baseline confounding and enable direct comparisons, patients with atrial fibrillation were matched in a 1:1 ratio with patients without atrial fibrillation. Matching was performed on sex, comorbidities, and major cardiovascular risk factors, yielding two equally sized groups of 118 patients. This approach provided a balanced study population and strengthened the validity of the subsequent analyses. Patients were then categorized into two groups: Group A comprised 118 individuals with paroxysmal atrial fibrillation, acute ischemic stroke, and cardiovascular risk factors, while Group B included 118 patients with acute ischemic stroke and CVRFs but without atrial fibrillation. All patients exhibited progressive ischemic stroke and had not been previously diagnosed with either atrial fibrillation or stroke.

**Figure 1:**
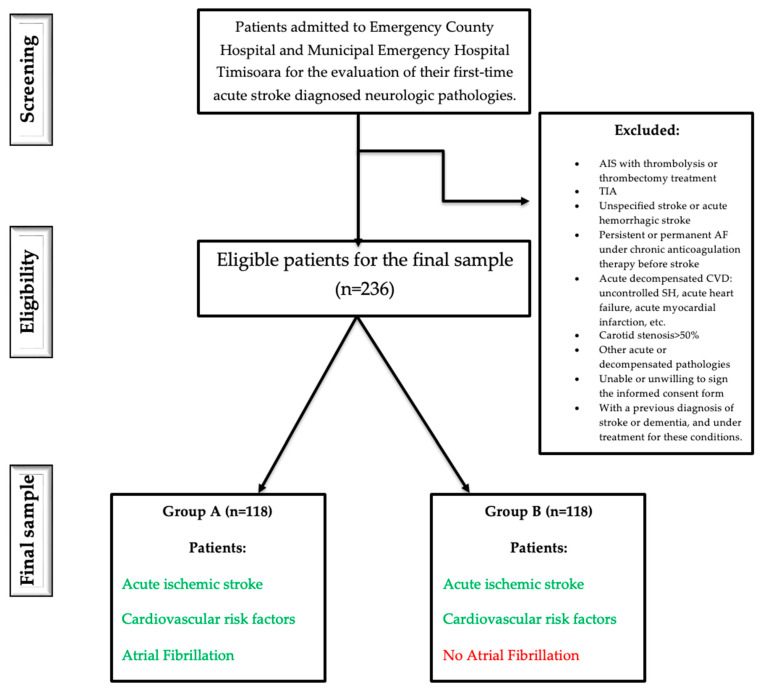
Flowchart study.

Additionally, none were receiving thrombolytic therapy, thrombectomy, or chronic anticoagulation treatment at the time of the stroke. Within Group A, the diagnosis of paroxysmal atrial fibrillation was made during hospitalization for the acute ischemic event; none of the patients were found to have persistent or permanent atrial fibrillation (Figure 1).

Inclusion criteria were: (1) age ≥ 18 years, (2) presence of CVRFs including carotid atheromatosis (defined as carotid plaque or carotid stenosis < 50%), (3) paroxysmal AF concurrent with a first-ever acute ischemic stroke and no history of oral anticoagulation therapy before the event, and (4) neuroimaging-confirmed acute ischemic stroke with no prior stroke history.

Exclusion criteria included: (1) ischemic stroke treated with thrombolysis or thrombectomy, (2) transient ischemic attack (TIA), unspecified stroke, or hemorrhagic stroke, (3) persistent or permanent AF with chronic anticoagulation, (4) acute decompensated cardiovascular conditions (e.g., uncontrolled systemic hypertension, acute myocardial infarction, or acute heart failure), (5) carotid stenosis > 50%, (6) presence of other acute or decompensated systemic diseases, (7) inability or unwillingness to provide informed consent, and (8) prior diagnosis or treatment for stroke or dementia. Patients with incomplete clinical or functional data were excluded from the final analyses. No imputation for missing values was performed.

### 2.2. Clinical and Cardiac Diagnostic Assessment

All patients underwent thorough evaluations encompassing clinical, neurological, and cardiological assessments. These included a detailed medical history, a physical examination, and heart rate measurement, systolic blood pressure (SBP), and diastolic blood pressure (DBP). Laboratory tests comprised lipid profiles along with evaluations of additional cardiovascular risk factors. Paroxysmal AF was diagnosed according to current European Society of Cardiology (ESC) guidelines [5,17], and defined as AF de novo diagnosed in emergency or during the hospitalization of the first acute ischemic stroke based on clinical symptoms and confirmation via resting 12-lead electrocardiogram (ECG) or 24 h Holter ECG monitoring.

In accordance with the 2020 European Society of Cardiology (ESC) guidelines [5], thromboembolic and hemorrhagic risks for patients experiencing paroxysmal atrial fibrillation and acute ischemic stroke were assessed utilizing the CHA_2_DS_2_-VASc and HAS-BLED scores. This study employed the CHA_2_DS_2_-VASc score as the primary method for evaluating thromboembolic risk, given that it was the endorsed and validated risk assessment tool recommended by the ESC guidelines during the data collection period [17].

### 2.3. Functional and Stroke Severity Assessment

All individuals participating in the study underwent obligatory cerebral computed tomography to verify the diagnosis of acute ischemic stroke. Furthermore, a resting electrocardiogram and carotid artery Doppler ultrasonography were conducted in every instance to detect carotid atheromatosis, characterized by carotid plaques or stenosis. On the seventh day following the stroke or at the time of discharge, stroke severity, motor impairments, level of disability, and functional independence in daily living activities were evaluated utilizing the following validated scales: the National Institutes of Health Stroke Scale (NIHSS) [18], the Medical Research Council (MRC) scale, the modified Rankin Scale (mRS) [19], and the Activities of Daily Living (ADL) scale [20].

Stroke severity was evaluated using the NIHSS within a week following the acute ischemic incident or upon hospital discharge. Each component is scored separately, and the cumulative NIHSS score—ranging from 0 to 42—represents the overall severity of neurological deficits, with elevated scores signifying more severe outcomes following a stroke [21,22].

The Medical Research Council scale is frequently utilized in stroke rehabilitation environments to measure changes in motor function [23]. The MRC scale employs manual muscle testing and rates strength on a six-point continuum from 0 to 5 [24].

The Activities of Daily Living (ADL) evaluates a patient’s capacity to execute essential self-care activities and ascertains their degree of functional independence, particularly among elderly individuals or those with disabilities [25]. This scale operates on a range from 0 to 10, with higher scores reflecting enhanced independence and functionality.

The modified Rankin Scale (mRS) was utilized to evaluate the functional status of patients either at discharge or seven days following admission. A threshold of mRS ≥3 was used to define moderate to severe disability [19].

### 2.4. Ethical Considerations

The study adhered to the principles outlined in the Declaration of Helsinki and received approval from the Institutional Review Boards of both participating hospitals: County Emergency Hospital Timisoara (approval no. 440 from 23 February 2024) and Municipal Emergency Hospital Timisoara (approval no. E1518 from 17 March 2021). All patients provided written informed consent before participation.

### 2.5. Statistical Analysis

Statistical analysis was performed utilizing IBM SPSS Statistics for Windows, version 20.0, with a significance threshold at *p* < 0.05. Categorical variables were represented as frequencies and percentages. In contrast, continuous variables were reported as means ± standard deviation. Unpaired t-tests were employed to compare continuous variables between groups, while chi-square tests were utilized for categorical variables. Spearman’s correlation coefficient was applied to evaluate relationships between variables, and the corresponding 95% confidence intervals (CI) for the correlation coefficients were calculated using the method proposed by Bonett and Wright [26].

Considering that the modified Rankin Scale served as a primary outcome, the statistical strength of this comparison was assessed utilizing G*Power 3.1.9.2 software. The average mRS values were determined to be 2.58 for group A (with AF) and 1.64 for group B (without AF), yielding an effect size of 0.94 for both groups (*n* = 118 for each). The calculated power reached 99.99%, surpassing the generally accepted benchmark of 80%, affirming the sample size’s sufficiency.

Logistic regression, applying the forward method grounded in the Wald test, was utilized to determine prognostic factors linked to post-stroke disability. The variables assessed encompassed mRS, Activities of Daily Living, age, NIHSS, and MRC scores for all stroke patients, in addition to CHA_2_DS_2_-VASc and HAS-BLED scores specifically for patients with atrial fibrillation (AF). The reported results included odds ratios (ORs) and their corresponding 95% confidence intervals (CIs).

Multivariable logistic regression was used to identify poor functional outcome predictors (mRS ≥ 3). For binary outcomes (e.g., mRS ≥ 3 vs. <3), logistic regression was specified as: logit(P(Y = 1)) = β0 + β1X1 + β2X2 + … + βkXk + ε. Where Y is the binary dependent variable, and X1 … Xk are the independent covariates.

For continuous outcomes (e.g., NIHSS or ADL scores), linear regression was applied as: Y = β0 + β1X1 + β2X2 + … + βkXk + ε. Where Y represents the continuous dependent variable, and X1 … Xk are the independent covariates.

The assessment of predictive model performance for continuous variables, including ADL (applicable to all patients and those with AF), NIHSS and MRC (considered across all patients as well as both AF and non-AF subgroups), and HAS-BLED (specific to AF patients), was conducted utilizing sensitivity, specificity, positive predictive value (PPV), and negative predictive value (NPV). Receiver operating characteristic (ROC) curves were constructed to evaluate diagnostic accuracy, and the area under the curve (AUC) was computed.

## 3. Results

### 3.1. Characteristics of the Study Population

As presented in Table 1, patients experiencing paroxysmal atrial fibrillation (AF) alongside acute ischemic stroke (Group A) exhibited notably poorer functional outcomes in comparison to those without AF (Group B). While both groups demonstrated comparable demographic characteristics and shared cardiovascular risk factors, including hypertension, diabetes, and chronic heart failure (all *p* > 0.05), a significant disparity was noted in the prevalence of carotid atheromatosis—100% in Group B contrasted with only 40.7% in Group A (*p* < 0.001). This finding emphasizes the differing mechanisms underlying stroke events (atherothrombotic versus cardioembolic). Furthermore, the incidence of disability, as assessed by the modified Rankin Scale (mRS ≥ 3), was significantly greater in the AF cohort (55.1% vs. 25.4%, *p* < 0.001), suggesting that strokes related to AF are more frequently linked to severe outcomes and increased dependency in daily living activities (Table 1).

### 3.2. Comparative Analysis of Age, Neurological Symptoms, Motor Deficit, Activity of Daily Living, and the Grade of Disability in Both Groups of Patients Included in the Study

The data show that patients with acute ischemic stroke and paroxysmal atrial fibrillation (Group A) were significantly older and had more severe strokes compared to those without atrial fibrillation (Group B), as indicated by higher NIHSS scores (8.56 vs. 5.32, *p* < 0.001). Additionally, Group A demonstrated worse functional outcomes, with lower muscle strength (MRC: 3.57 vs. 4.15, *p* < 0.001), reduced independence in daily activities (ADL: 3.03 vs. 4.26, *p* = 0.005), and greater overall disability (mRS: 2.58 vs. 1.64, *p* < 0.001). These findings suggest that paroxysmal AF in the context of acute ischemic stroke is associated with older age, more severe neurological deficits, and poorer functional status at presentation (Table 2).

### 3.3. Correlation Between mRS, NIHSS, MRC, ADL, and Age in All Patients

In patients with acute ischemic stroke (*n* = 236), NIHSS scores showed a strong negative correlation with MRC (r = −0.915, *p* < 0.001), indicating that higher stroke severity is associated with reduced muscle strength. NIHSS also had a moderate negative correlation with ADL (r = −0.312, *p* < 0.001), and a strong positive correlation with mRS (r = 0.824, *p* < 0.001), reflecting poorer functional status and greater disability in more severe strokes. There was no significant correlation between NIHSS and age (r = 0.018, *p* = 0.788). The mRS also correlated negatively with MRC (r = −0.794) and ADL (r = −0.282), and positively, though weakly, with age (r = 0.184, *p* = 0.005). Age had no significant relationship with MRC or ADL. Finally, a weak but significant positive correlation was observed between MRC and ADL (r = 0.287, *p* < 0.001). These findings highlight that stroke severity and muscle strength are key drivers of disability and independence post-stroke, while age has a more limited impact (Table 3).

### 3.4. Correlation Analysis in Stroke Patients with Paroxysmal Atrial Fibrillation (Group A)

In patients with acute ischemic stroke and paroxysmal atrial fibrillation (Group A), a very strong negative correlation was identified between NIHSS scores and MRC (r = −0.932), as well as a moderate negative correlation with ADL (r = −0.414). A strong positive correlation was also observed between NIHSS and mRS (r = 0.795). While age did not exhibit a significant correlation with NIHSS, MRC, or ADL, it demonstrated a weak positive correlation with mRS (r = 0.186, *p* = 0.044), implying a minor influence of age on disability. Furthermore, MRC and ADL were positively correlated (r = 0.375, *p* < 0.001) (Table 4).

In paroxysmal AF and CVRFs patients (group A), regarding CHA_2_DS_2_-VASc values, they correlated very weak negatively but statistically significant with ADL (r = −0.190, 95% CI −0.360; −0.008, *p* = 0.039), and weak positively statistically substantial with age (r = 0.375, 95% CI 0.202; 0.525, *p* = 0.000) and strong positively statistically significant with HAS-BLED (r = 0.635, 95% CI 0.500; 0.740, *p* = 0.000).

Furthermore, in patients with acute ischemic stroke and paroxysmal atrial fibrillation (Group A), a weak yet statistically significant positive correlation was identified between the HAS-BLED score and NIHSS (r = 0.190, *p* = 0.039). This finding suggests that a higher risk of bleeding is marginally associated with more severe strokes. Additionally, HAS-BLED exhibited weak negative correlations with both MRC and ADL (r = −0.174); however, these correlations did not achieve statistical significance (*p* = 0.059), indicating a potential trend towards diminished muscle strength and reduced independence in patients at greater bleeding risk. A similarly weak and non-significant positive correlation was observed with mRS (r = 0.165, *p* = 0.074), hinting at a possible association with increased disability. No significant correlation was found between HAS-BLED and age (r = 0.045, *p* = 0.629). These results imply that while the HAS-BLED score may provide a weak indication of stroke severity and functional impairment, its predictive capacity within this context remains limited (Table 5).

### 3.5. Evaluation of Prognostic Factors on Moderate to Severe Disability (mRS ≥ 3) in All Acute Stroke Patients

Binary logistic regression analysis was conducted to determine independent predictors of moderate-to-severe disability (mRS ≥ 3 at discharge). Three distinct models were developed, considering clinical relevance and subgroup analysis.

#### 3.5.1. Model 1—ADL and Age

Logistic regression analysis revealed statistically significantly lower odds of mRS ≥ 3 for an increase in activity of daily living (ADL score) when considering all acute ischemic stroke patients (*p* < 0.001). Also, it revealed statistically significantly lower odds of mRS > 3 for an increase in activity of daily living (ADL score) when considering paroxysmal AF patients with acute ischemic stroke (group A) (*p* < 0.001). In this logistic regression model with ADL and age, age was not found to be significant in all acute ischemic stroke patients, nor in the 118 paroxysmal AF patients with acute ischemic stroke (group A). In patients without AF and with stroke (group B), ADL and age were not statistically significant in the final logistic model. (Table 6).

**Figure 2:**
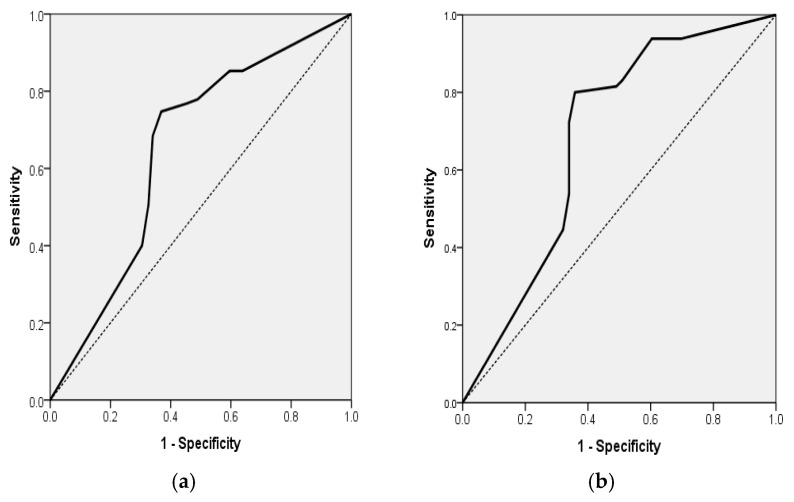
(**a**) Receiver Operating Characteristic (ROC) curve for a multiple logistic regression model for the association of mRS ≥ 3 for all 236 patients with acute ischemic stroke with the ADL independent variable (AUROC = 0.649, 95% CI 0.578; 0.720, *p* < 0.001). (**b**) Receiver Operating Characteristic (ROC) curve for multiple logistic regression model for the association of mRS ≥ 3 for the 118 AF patients with acute ischemic stroke with ADL independent variable (AUROC = 0.682, 95% CI 0.580; 0.784, *p* = 0.001).

For the entire sample (*n* = 236), the final model accounted for 12.2% of the variance in disability (Nagelkerke R^2^) and accurately classified 60.59% of the cases. ADL was identified as an independent protective factor; specifically, a 1-point increase in the ADL score corresponded to a 17.8% reduction in the odds of experiencing moderate-to-severe disability (mRS ≥ 3) (OR = 0.822; 95% CI, 0.756–0.895; *p* < 0.001). In addition, the probability of mRS ≥ 3 for all 236 patients with acute ischemic stroke can be estimated by the formula: exp(RS)/(1 + exp(RS)), where RS = −0.195 + 0.272x (ADL). This model had a sensitivity of 50.53%, specificity of 67.38%, positive predictive value (PPV) of 51.06% and negative predictive value (NPV) of 66.90%. The area under the ROC curve for this final model was 0.649 (AUROC = 0.649, 95% CI 0.578–0.720, *p* < 0.001). (Table 6 and Figure 2a).

In group 1, which comprised AF patients (*n* = 118), the final model demonstrated significance (*p* < 0.001), with a corresponding Nagelkerke R^2^ = 0.181, achieving a classification accuracy of 72.88%. The ADL score continued to serve as a robust predictor, with each 1-point increment correlating with a 22.7% decrease in the odds of mRS ≥ 3 (OR = 0.773; 95% CI, 0.680–0.879; *p* < 0.001). In addition, the probability of mRS ≥ 3 for the 118 AF patients with acute stroke can be estimated by the formula: exp(RS)/(1 + exp(RS)), where RS = −0.257 + 0.998x (ADL). This model had a sensitivity of 80%, specificity of 64.15%, positive predictive value (PPV) of 73.24%, and negative predictive value (NPV) of 72.34%. The area under the ROC curve for this final model was 0.682 (AUROC = 0.682, 95% CI 0.580–0.784, *p* = 0.001) (Table 6 and Figure 2b).

In group 1, which comprised AF patients (*n* = 118), the model demonstrated significance (χ^2^ = 35.02, df = 2, *p* < 0.001; Nagelkerke R^2^ = 0.325), achieving a classification accuracy of 72.0%. The ADL score continued to serve as a robust predictor, with each 1-point increment correlating with a 22.7% decrease in the odds of mRS ≥ 3 (OR = 0.773; 95% CI, 0.680–0.879; *p* < 0.001). Again, Age was not a significant predictor (OR = 1.017; 95% CI, 0.982–1.053; *p* = 0.331) (Table 6 and Figure 2b).

#### 3.5.2. Model 2—NIHSS and MRC

**Figure 3:**
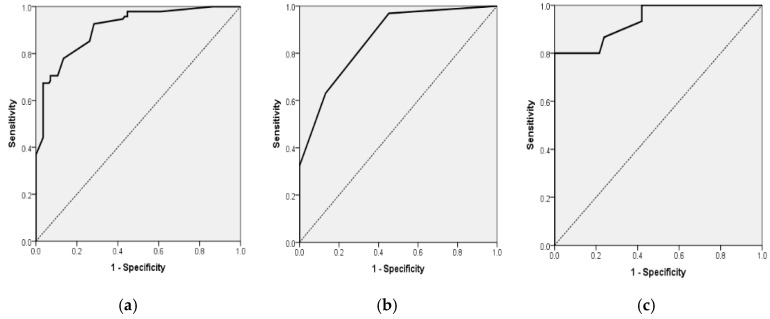
(**a**). Receiver Operating Characteristic (ROC) curve for multiple logistic regression model for the association of mRS ≥ 3 for all 236 patients with acute stroke with NIHSS and MRC scales as independent variables (AUROC = 0.908, 95% CI 0.870; 0.945, *p* < 0.001). (**b**). Receiver Operating Characteristic (ROC) curve for multiple logistic regression model for the association of mRS ≥ 3 for the 118 AF patients with acute stroke (group A) with MRC scale as independent variables (AUROC = 0.858, 95% CI 0.793; 0.924, *p* < 0.001). (**c**). Receiver Operating Characteristic (ROC) curve for multiple logistic regression model for the association of mRs ≥ 3 for 118 patients with acute stroke without AF (group B) with NIHSS and MRC scales as independent variables (AUROC = 0.935, 95% CI 0.883; 0.987, *p* < 0.001).

In the total cohort (*n* = 236), the logistic regression model that incorporated NIHSS and MRC scores demonstrated statistical significance (χ^2^ = 91.58, df = 2, *p* < 0.001), accounting for 48.4% of the variance in disability (Nagelkerke R^2^) and achieving a classification accuracy of 80.9%. An increase in NIHSS scores was associated with a 31.6% rise in the odds of moderate-to-severe disability for each additional point (OR = 1.316; 95% CI, 1.097–1.579; *p* = 0.003). Conversely, higher MRC scores provided a protective effect, decreasing the odds by 64.5% per point (OR = 0.355; 95% CI, 0.151–0.834; *p* = 0.017) (Table 7 and Figure 3a).

Logistic regression analysis revealed statistically significantly higher odds of mRS ≥ 3 for an increase in hemorrhagic risk (HASBLED score increased by 1 unit) in paroxysmal AF acute stroke patients. CHA_2_DS_2_-VASc was not found to be statistically significant in this model in paroxysmal AF patients with acute stroke (group A), regarding the prognosis of moderate to severe disability (mRS ≥ 3). This model was made only for paroxysmal AF and acute ischemic stroke patients (group A), as in patients with acute stroke without AF (group B), CHA_2_DS_2_-VASc and HAS-BLED were not calculated.

In patients diagnosed with paroxysmal atrial fibrillation, the model demonstrated a high level of significance (χ^2^ = 73.44, df = 2, *p* < 0.001; Nagelkerke R^2^ = 0.551) and achieved a classification accuracy of 82.2%. Within this subgroup, MRC emerged as the sole independent predictor, with each additional point correlating to an 83.5% decrease in the odds of mRS ≥ 3 (OR = 0.165; 95% CI, 0.085–0.321; *p* < 0.001). The NIHSS did not show significance (OR = 1.172; 95% CI, 0.897–1.529; *p* = 0.245) (Table 7 and Figure 3b).

In the cohort of patients without atrial fibrillation, the model demonstrated statistical significance (χ^2^ = 28.64, df = 2, *p* < 0.001; Nagelkerke R^2^ = 0.360), achieving a classification accuracy of 79.5%. Within this population, an increase in NIHSS scores correlated with a 43.4% rise in the likelihood of disability for each additional point (OR = 1.434; 95% CI, 1.069–1.923; *p* = 0.016), whereas higher MRC scores were associated with a 75.8% decrease in the odds per point (OR = 0.242; 95% CI, 0.064–0.913; *p* = 0.036) (Table 7 and Figure 3c).

#### 3.5.3. Model 3—HAS-BLED and CHA_2_DS_2_-VASc (Patients with Paroxysmal Atrial Fibrillation Only)

Logistic regression analysis revealed statistically significantly higher odds of mRS ≥ 3 for an increase in hemorrhagic risk (HASBLED score increased by 1 unit) in paroxysmal AF acute stroke patients. CHA_2_DS_2_-VASc was not found to be statistically significant in this model in paroxysmal AF patients with acute stroke (group A), regarding the prognostic of moderate to severe disability (mRS ≥ 3). This model was made only for paroxysmal AF and acute ischemic stroke patients (group A), as in patients with acute stroke without AF (group B), CHA_2_DS_2_-VASc and HASBLED were not calculated.

**Figure 4:**
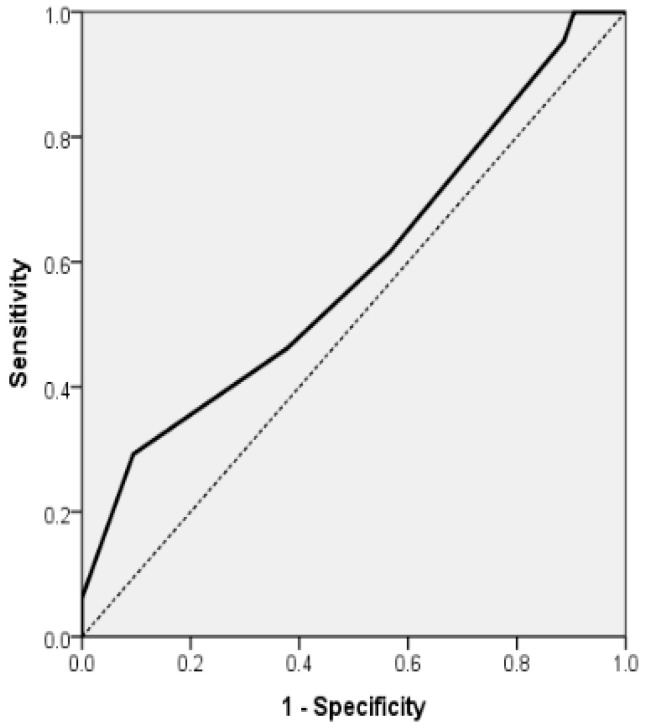
Receiver Operating Characteristic (ROC) curve for multiple logistic regression model for the association of mRS ≥ 3 for the 118 AF patients with acute stroke (group A) and with HAS-BLED scale as independent variables (AUROC = 0.589, 95% CI 0.487; 0.692, *p* = 0.096).

In patients with paroxysmal atrial fibrillation, the final logistic regression model incorporating HAS-BLED demonstrated statistical significance (*p* = 0.011), accounted for 5.0% of the variance (Nagelkerke R^2^) and achieved a classification accuracy of 53.39%. An increase of 1 point in the HAS-BLED score was found to be independently linked to a heightened risk of disability, with the odds of mRS ≥ 3 increasing by 32.5% (OR = 1.325; 95% CI, 1.015–1.729; *p* = 0.039). In addition, probability of mRS ≥ 3 for the 118 AF patients with acute stroke (group A) can be estimated by the formula: exp(RS)/(1 + exp(RS)), where RS = −0.670 + 0.281x (HASBLED). The model had a sensitivity of 61.54%, specificity of 43.40%, positive predictive value (PPV) of 57.14% and negative predictive value (NPV) of 47.92%. The area under the ROC curve for this final model was 0.589 (AUROC = 0.589, 95% CI 0.487–0.692, *p* = 0.096) (Table 8 and Figure 4).

## 4. Discussion

This study investigated the predictive significance of clinical and functional variables concerning post-stroke disability, with an emphasis on the distinctions between patients with atrial fibrillation and those without. Using logistic regression models, we established that functional indicators—Activities of Daily Living and Medical Research Council muscle strength scores—serve as robust, independent predictors of unfavorable outcomes. In contrast, the National Institutes of Health Stroke Scale displayed a fluctuating predictive capacity contingent upon atrial fibrillation.

Also, we analyzed the clinical characteristics and predictors of functional outcomes in patients experiencing acute ischemic stroke, both with and without paroxysmal atrial fibrillation. The results indicate that individuals with paroxysmal atrial fibrillation were generally older and exhibited more severe neurological deficits upon admission, diminished muscle strength, reduced functional independence, and increased disability scores in comparison to their counterparts without atrial fibrillation. Importantly, our study revealed a significantly higher prevalence of carotid atheromatosis among patients without atrial fibrillation, highlighting the distinct stroke mechanisms associated with cardioembolic versus large-artery atherosclerotic etiologies [27].

In our research, we found that patients suffering from acute ischemic stroke and paroxysmal atrial fibrillation who also presented with cardiovascular risk factors demonstrated significantly poorer functional outcomes (mRS ≥ 3) when compared to those without AF. This disparity occurred despite no notable differences in the prevalence of conditions such as hypertension, dyslipidemia, diabetes mellitus, chronic coronary syndrome, chronic heart failure, smoking, or alcohol consumption between the two groups. Interestingly, carotid atherosclerosis was found to be more prevalent among patients without AF, indicating that large-artery disease may play a more significant role in the etiology of stroke within this subgroup. At the same time, cardioembolism could be more pertinent in strokes associated with AF. These results are somewhat consistent with the findings of Gorczyca-Głowacka et al. [28], who indicated that ischemic stroke patients with AF tended to be older, more frequently female, and exhibited higher rates of hypertension, ischemic heart disease, heart failure, and previous stroke/transient ischemic attack compared to those in sinus rhythm. Conversely, lifestyle risk factors such as smoking and alcohol consumption were more prevalent among the sinus rhythm cohort. Both studies reported increased stroke severity and a poorer short-term prognosis in patients with AF, corroborating previous literature that links AF with more debilitating strokes [28,29,30]. However, Gorczyca-Głowacka et al. study [28] revealed no notable difference in the prevalence of significant ICA stenosis between groups with atrial fibrillation and those with sinus rhythm. This underscores a potential contrast to our findings of increased carotid atherosclerosis in patients without AF. This discrepancy may be attributed to differences in patient selection, the type of AF, or variations in regional patterns of vascular disease. Collectively, these findings support the notion that, although AF is consistently associated with heightened disability in the context of acute ischemic stroke, the vascular profiles and stroke mechanisms may vary significantly between AF and non-AF populations. This highlights the necessity for customized secondary prevention strategies [28,31].

We observed that patients with paroxysmal AF and CVRFs presenting with acute ischemic stroke were older, had more pronounced neurological deficits (higher NIHSS scores), greater motor impairment (lower MRC scores), poorer daily activity performance (lower ADL scores), and higher disability risk (higher mRS) compared to those without AF. Increasing age in AF patients was associated with greater disability (higher mRS), while improved motor function (higher MRC) and ADL were linked to lower disability scores. These results align with extensive studies, such as those conducted by Vinding et al. [29], which revealed that AF patients experience nearly double the incidence of very severe strokes (NIHSS > 14) relative to non-AF patients, with post-stroke mortality being more closely tied to the severity of the stroke than to the presence of AF itself. The mechanisms underlying the poorer outcomes in strokes related to AF may include factors such as diminished cardiac output, larger infarct sizes, reduced collateral circulation, and an elevated risk of hemorrhagic transformation [32]. Data at the population level from Denmark suggest a temporal reduction in severe strokes, likely attributable to enhanced management of CVRFs, advancements in imaging techniques, the establishment of specialized stroke units, and increased use of anticoagulants [32]. Nevertheless, AF remains associated with more severe clinical presentations [29].

Similarly to our results, data from the Canadian Stroke Network [33] revealed that atrial fibrillation (AF) correlates with heightened disability and mortality; however, a significant portion of this effect diminished after adjustments for age and stroke severity were made, highlighting the prognostic relevance of these variables. Age and the National Institutes of Health Stroke Scale (NIHSS) score [34], are strong predictors of functional outcomes following a stroke, with NIHSS in the acute phase demonstrating robust associations with the modified Rankin Scale (mRS) during follow-up [35] and with other disability assessments, including the Barthel Index and IADL [36,37]. Additionally, comorbidities such as depression [38] and chronic heart failure [39] may further aggravate functional deterioration and should be consistently evaluated. Taken together, our results and existing literature suggest that in strokes related to AF, disability outcomes are predominantly influenced by age, initial neurological severity, and functional status rather than by AF alone, underscoring the necessity for thorough assessment and focused rehabilitation approaches.

Our research indicates a strong correlation between enhancements in ADL and a reduction in disability following an acute ischemic stroke, particularly among patients experiencing paroxysmal atrial fibrillation (AF). Notably, an increase of one unit in the ADL score corresponds to a 22.7% reduction in the likelihood of moderate to severe disability (mRS ≥ 3) for those with AF (OR = 0.773, 95% CI 0.680–0.879, *p* < 0.001), compared to a 17.8% decrease observed in the overall stroke population. This finding is in agreement with previous studies that have shown functional recovery is influenced by various factors, including the type of ischemic stroke, lower initial severity, younger age, and intensive rehabilitation efforts, such as home-based programs [40,41,42]. Additionally, the risks associated with cognitive decline and disability are influenced by ADL performance, as well as cardiovascular comorbidities and depression [43]. Furthermore, neurological severity, measured by the NIH Stroke Scale (NIHSS), emerged as a significant factor affecting disability within our cohort, with each incremental increase in NIHSS score elevating the odds of mRS ≥ 3 by 31.6% (OR = 1.316, 95% CI 1.097–1.579, *p* < 0.05). This observation aligns with numerous studies that indicate higher NIHSS scores are predictive of poorer functional outcomes, increased mortality rates, and diminished benefits from recanalization following endovascular interventions [44,45,46]. The severity of motor deficits, as quantified by the MRC scale, exhibited the strongest correlation with functional independence. Each additional unit of motor deficit was associated with a 64.5% reduction in the odds of mRS ≥ 3 within the overall cohort and an 83.5% reduction among patients with atrial fibrillation (AF), highlighting the crucial importance of motor recovery in influencing post-stroke outcomes. These results are corroborated by findings indicating that interventions such as targeted motor training, physical therapy, and neuroplasticity-based approaches (including mirror therapy and non-invasive brain stimulation) enhance recovery trajectories; however, it is important to note that not all patients adhere to the proportional recovery rule [47,48,49]. Overall, our findings indicate that in strokes related to AF, advancements in activities of daily living (ADL) and motor function confer a significantly greater protective effect against long-term disability when compared to non-AF strokes. This suggests that tailored rehabilitation strategies emphasizing early mobilization, motor training, and enhancing daily functions may benefit this high-risk subgroup most substantially.

In group A, we found that CHA_2_DS_2_-VASc correlated weakly and inversely with ADL (*p* < 0.05) and positively with age (*p* < 0.001) and HAS-BLED (*p* < 0.001), while HAS-BLED correlated weakly and positively with NIHSS (*p* < 0.05). A strong positive correlation between CHA_2_DS_2_-VASc and HAS-BLED suggests that as new CVRFs or cardiovascular/cerebrovascular events accrue, both functional independence declines and hemorrhagic risk increase. These results support prior findings that stroke and bleeding risks in AF are dynamic over time, requiring periodic reassessment for accurate prognostication [50]. Within our AF cohort, each one-unit increase in HAS-BLED was linked to a 32.5% rise in the odds of experiencing moderate to severe disability (mRS ≥ 3; OR = 1.325, 95% CI 1.015–1.729, *p* < 0.05), aligning with evidence that higher CHA_2_DS_2_-VASc scores are indicative of an increased incidence of stroke and mortality over a 3–5 year period [51].

Across our entire study population, neurological severity at admission (NIHSS) was a strong predictor of disability, with each additional point increasing the odds of mRS ≥ 3 by 31.6%. In contrast, motor strength (MRC) was a potent protective factor—each additional unit reduced the odds of mRS ≥ 3 by 64.5%. Notably, in AF patients, the protective effect of motor recovery was even greater, with an 84.5% reduction in the odds of mRS ≥ 3 for each MRC point gained, compared with 75.8% in non-AF patients. This reinforces the critical role of targeted motor rehabilitation in AF-related stroke, a group in which gains in motor strength appear to translate more efficiently into disability reduction. These results are consistent with previous research that emphasizes the NIHSS as a strong predictor of functional outcomes following a stroke [52] and indicate that while improvements in motor function are crucial, they do not always directly correlate with favorable mRS outcomes.

Another relevant aspect is the potential difference in lesion localization between AF-related and non-AF strokes. Cardioembolic strokes, which are more frequent in AF, often present as large cortical infarcts in the middle cerebral artery territory, whereas non-AF strokes due to large-artery atherosclerosis or small-vessel disease more commonly involve subcortical or lacunar lesions. Previous imaging studies have shown that AF patients tend to develop larger and more disabling infarcts, frequently with cortical involvement, which may explain the more severe neurological deficits and poorer functional outcomes observed in our cohort [53,54]. In contrast, the higher prevalence of carotid atheromatosis in the non-AF group may predispose these patients to territorial infarcts of varying size or lacunar patterns, typically associated with less severe early disability [55]. Future studies incorporating systematic imaging analysis of lesion localization and volume are warranted to better clarify how stroke topography mediates the prognostic impact of AF, particularly its paroxysmal form.

To our knowledge, this is among the first studies to specifically examine short-term disability trajectories in patients with paroxysmal AF after acute ischemic stroke. While previous research has generally grouped all AF types together, our findings highlight that even paroxysmal AF is associated with significantly greater neurological severity and functional dependence compared to non-AF patients. In addition, by integrating traditional cardiovascular risk scores (CHA_2_DS_2_-VASc, HAS-BLED) with disability scales (NIHSS, MRC, ADL, mRS), this study provides new insights into the interplay between cardiac comorbidity and functional prognosis. These contributions expand upon earlier studies and emphasize the need to consider paroxysmal AF not merely as a transient arrhythmia, but as a clinically relevant determinant of early post-stroke disability.

## 5. Study Limitations and Strengths

It is important to recognize several limitations inherent in our study. Firstly, the research was conducted at a single center, which may restrict the applicability of our findings to other populations exhibiting different demographic and clinical characteristics. Secondly, while the sample size was relatively balanced between the AF and non-AF groups, it was modest, potentially diminishing the statistical power to identify minor differences between groups regarding less prevalent variables. Thirdly, patients with AF were categorized based on a diagnosis of paroxysmal AF at presentation, and continuous cardiac monitoring was not conducted for all individuals; consequently, it is possible that undetected paroxysmal AF episodes occurred in the non-AF group, leading to potential misclassification bias. Fourthly, although we accounted for major cardiovascular risk factors, residual confounding remains due to unmeasured variables such as socioeconomic status, medication adherence, anticoagulation quality, or genetic predisposition. Fifthly, functional outcomes (mRS, ADL, MRC) were evaluated during the acute hospitalization phase, and no long-term follow-up was conducted (up to 7 days or discharge). Without long-term follow-up, we cannot determine how paroxysmal AF influences chronic disability trajectories, recovery patterns, or recurrent events. This limitation highlights the need for multicenter prospective studies incorporating prolonged rhythm monitoring and extended follow-up to provide a more complete understanding of prognosis. Lastly, the diagnosis of carotid atherosclerosis was based on ultrasound findings, and inter-observer variability was not formally evaluated, which may introduce measurement bias. Furthermore, while correlations among CHA_2_DS_2_-VASc, HAS-BLED, NIHSS, and MRC offer valuable prognostic insights, their predictive value may be affected by the dynamic nature of clinical changes post-stroke, and repeated longitudinal assessments would yield a more comprehensive risk profile.

This study has several notable strengths. Firstly, we examined a clearly defined and equally sized cohort of acute ischemic stroke patients, both with and without paroxysmal atrial fibrillation, all of whom shared cardiovascular risk factors. This design facilitated direct comparisons while minimizing baseline confounding from significant vascular comorbidities. Secondly, the research incorporated structural vascular assessments, specifically carotid atherosclerosis, and various validated clinical scales (mRS, NIHSS, ADL, MRC), enabling a comprehensive evaluation of functional outcomes. Thirdly, our investigation uniquely assessed the relationships between CHA_2_DS_2_-VASc, HAS-BLED, and functional status, providing novel insights into how these widely utilized risk scores correlate with the severity of disability in strokes related to AF. Fourthly, we measured the differential effects of motor recovery (MRC improvement) on disability reduction in patients with AF compared to those without, revealing a potentially greater benefit in the AF cohort. This observation carries significant implications for rehabilitation. Fifthly, we situated our findings within the context of previous large-scale studies’ results, thereby reinforcing our conclusions’ external relevance. Lastly, the precise statistical reporting utilized, including odds ratios and confidence intervals, bolsters our findings’ reproducibility and clinical applicability.

## 6. Conclusions

Our findings from our single-center study suggest that paroxysmal AF is associated with greater neurological severity and poorer short-term functional outcomes after acute ischemic stroke. However, the absence of continuous ECG monitoring and long-term follow-up limits the certainty of these conclusions. The degree of post-stroke disability in these patients is strongly linked to age, ADL, MRC, stroke severity (based on NIHSS), HAS-BLED score, and overall disability level.

In paroxysmal AF patients, we found a direct association between the risk of moderate to severe disability and reduced ADL, worsening neurological symptoms, greater motor deficit, and advanced age. CHA_2_DS_2_-VASc scores correlated with ADL, while HAS-BLED scores were associated with NIHSS. Identifying patients at risk for moderate to severe disability is critical to preventing progression and improving outcomes, with prognostic factors closely tied to ADL, motor function, and hemorrhagic risk. Moreover, a multidisciplinary approach is crucial to designing tailored rehabilitation programs aimed at reducing disability, enhancing daily functioning, and promoting reintegration into society.

Future research should focus on the benefits of AF screening with extended monitoring and longitudinal evaluation, the role of AF-associated comorbidities in stroke outcomes, and the development of optimized recovery strategies specific to stroke patients with AF.

## Figures and Tables

**Table 1:** Demographic data for all patients and in patients with AF and acute ischemic stroke (group A) versus patients with acute ischemic stroke without AF (group B).

Variables	Total Sample (*n* = 236)	Group A (*n* = 118)	Group B (*n* = 118)	*p*
Mens	125 (53.00%)	60 (50.80%)	65 (55.10%)	0.514
Womens	111 (47.00%)	58 (49.20%)	53 (44.90%)	0.514
Hypertension	195 (82.60%)	93 (78.80%)	102 (86.40%)	0.122
Hyperlipemia	82 (34.70%)	40 (33.90%)	42 (35.60%)	0.785
Smoking	13 (5.50%)	8 (6.80%)	5 (4.20%)	0.790
Alcohol	15 (6.40%)	8 (6.80%)	7 (5.90%)	0.582
Diabetes mellitus	70 (29.70%)	39 (33.10%)	31 (26.30%)	0.254
Chronic coronary syndrome (CCS)	32 (13.60%)	17 (14.40%)	15 (12.70%)	0.704
Chronic Heart Failure (CHF)	132 (55.90%)	68 (57.60%)	64 (54.20%)	0.600
Carotid atheromatosis	166 (70.30%)	48 (40.70%)	118 (100%)	<0.001
mRS ≥ 3	95 (40.25%)	65 (55.08%)	30 (25.42%)	<0.001
mRS < 3	141 (59.70%)	53 (44.90%)	88 (74.60%)	<0.001

Legend: AF—Atrial Fibrillation, mRS—modified Rankin Scale, CCS—Chronic coronary syndrome, CHF—Chronic Heart Failure.

**Table 2:** Evaluation of Age, NIHSS, MRC, ADL, and mRS in All Patients with Acute Ischemic Stroke. Comparison between Group A (with Paroxysmal AF) and Group B (without AF).

Variables	Total Sample (*n* = 236)	Group A (*n* = 118)	Group B (*n* = 118)	*p*
Mean age	71.06 ± 10.20	73.34 ± 8.78	68.77 ± 11.01	0.001
NIHSS	6.94 ± 5.52	8.56 ± 6.09	5.32 ± 4.35	<0.001
MRC	3.86 ± 1.13	3.57 ± 1.27	4.15 ± 0.89	<0.001
ADL	3.65 ± 3.36	3.03 ± 3.14	4.26 ± 3.47	0.005
mRS	2.11 ± 1.38	2.58 ± 1.47	1.64 ± 1.10	<0.001

Legend: AF—Atrial Fibrillation; NIHSS—National Institutes of Health Stroke Scale; MRC—Medical Research Council; ADL—Activities of Daily Living scale; mRS—modified Rankin Scale.

**Table 3:** Correlation Between NIHSS, MRC, ADL, mRS, and Age in Acute Ischemic Stroke.

Parameter	MRC	ADL	mRS	Age
NIHSS				
R	−0.915	−0.312	0.824	0.018
95% CI	−0.937; −0.886	−0.425; −0.189	0.770; 0.866	−0.110; 0.145
*p*	<0.001	˂0.001	˂0.001	0.788
**Parameter**	**MRC**	**ADL**	**Age**	
mRS				
R	−0.794	−0.282	0.184	
95% CI	−0.842; −0.733	−0.398; −0.158	0.057; 0.306	
*p*	<0.001	˂0.001	0.005	
**Parameter**	**MRC**	**ADL**		
Age				
R	−0.108	−0.117		
95% CI	−0.233; 0.020	−0.242; 0.011		
*p*	0.099	0.074		
**Parameter**	**ADL**			
MRC				
R	0.287			
95% CI	0.163; 0.402			
*p*	<0.001			

Legend: NIHSS—National Institutes of Health Stroke Scale; MRC—Medical Research Council; ADL—Activities of Daily Living scale; mRS—modified Rankin.

**Table 4:** Correlation between NIHSS, MRC, ADL, mRS, and age in 118 acute ischemic stroke patients with paroxysmal AF (group A).

**Parameter**	**MRC**	**ADL**	**mRS**	**Age**
NIHSS				
R	−0.932	−0.414	0.795	0.087
95% CI	−0.956; −0.897	−0.559; −0.245	0.704; 0.860	−0.096; 0.264
*p*	<0.001	˂0.001	˂0.001	0.349
**Parameter**	**MRC**	**ADL**	**Age**	
mRS				
R	−0.786	−0.381	0.186	
95% CI	−0.854; −0.692	−0.530; −0.209	0.004; 0.356	
*p*	<0.001	˂0.001	0.044	
**Parameter**	**MRC**	**ADL**		
Age				
R	−0.120	−0.128		
95% CI	−0.295; 0.063	−0.302; 0.055		
*p*	0.196	0.167		
**Parameter**	**ADL**			
MRC				
R	0.375			
95% CI	0.202; 0.525			
*p*	<0.001			

Legend: NIHSS—National Institutes of Health Stroke Scale; MRC—Medical Research Council; ADL—Activities of Daily Living scale; mRS—modified Rankin.

**Table 5:** Correlation between CHA_2_DS_2_-VASc, HAS-BLED, NIHSS, MRC, ADL, mRS, and age for group A.

**Parameter**	**MRC**	**NIHSS**	**ADL**	**mRS**	**Age**	**HAS-BLED**
CHA_2_DS_2_-VASc						
R	0.028	0.010	−0.190	0.098	0.375	0.635
95% CI	−0.154; 0.208	−0.171; 0.190	−0.360; −0.008	−0.085; 0.274	0.202; 0.525	0.500; 0.740
*p*	0.766	0.918	0.039	0.293	<0.001	<0.001
**Parameter**	**MRC**	**NIHSS**	**ADL**	**mRS**	**Age**	
HAS-BLED						
R	−0.174	0.190	−0.174	0.165	0.045	
95% CI	−0.345; 0.008	0.008; 0.360	−0.345; 0.008	−0.017; 0.337	−0.137; 0.224	
*p*	0.059	0.039	0.059	0.074	0.629	

Legend: NIHSS—National Institutes of Health Stroke Scale; MRC—Medical Research Council; ADL—Activities of Daily Living scale; mRS—modified Rankin Scale; CHA_2_DS_2_-VASc—Score for Atrial Fibrillation Stroke Risk; HAS-BLED—Score for Hemorrhagic Risk Evaluation in Atrial Fibrillation.

**Table 6:** Multiple logistic regression analysis for risk of mRS ≥ 3 in all 236 patients with acute ischemic stroke and in 118 paroxysmal AF patients with acute ischemic stroke (group A), (ADL scale and age in the model).

Variable	Patients with mRS ≥ 3	OR (95% CI)	*p*-Value
**All 236 patients with acute ischemic stroke**			
ADL	95 (40.25%)	0.822 (0.756; 0.895)	<0.001
**118 paroxysmal AF patients with acute ischemic stroke (group A)**			
ADL	65 (55.08%)	0.773 (0.680; 0.879)	<0.001

Legend: AF—Atrial Fibrillation; ADL—Activities of Daily Living scale; mRS—modified Rankin Scale; OR = odds ratio, 95% CI = 95% confidence interval.

**Table 7:** Multiple logistic regression analysis for risk of mRS ≥ 3 in all 236 patients with acute ischemic stroke, in 118 paroxysmal AF patients with acute ischemic stroke (group A), and 118 patients with acute ischemic stroke without AF (group B) (NIHSS and MRC scales in the model).

Variable	Patients with mRS ≥ 3	OR (95% CI)	*p*-Value
**All patients with acute ischemic stroke**			
NIHSS	95 (40.25%)	1.316 (1.097; 1.579)	0.003
MRC	95 (40.25%)	0.355 (0.151; 0.834)	0.017
**118 paroxysmal AF patients with acute ischemic stroke (group A)**			
MRC	65 (55.08%)	0.165 (0.085; 0.321)	<0.001
**118 patients with acute ischemic stroke without AF (group B)**			
NIHSS	30 (25.42%)	1.434 (1.069; 1.923)	0.016
MRC	30 (25.42%)	0.242 (0.064; 0.913)	0.036

Legend: AF—Atrial Fibrillation; NIHSS—National Institutes of Health Stroke Scale; MRC—Medical Research Council; mRS—modified Rankin Scale; OR = odds ratio, 95% CI = 95% confidence interval.

**Table 8:** Multiple logistic regression analysis for risk of mRS > 3 in the 118 paroxysmal AF patients with acute ischemic stroke (group A) (CHA_2_DS_2_-VASc and HAS-BLED in the model).

Variable	Patients with mRS ≥ 3	OR (95% CI)	*p*-Value
**118 paroxysmal AF patients with acute ischemic stroke (group A)**			
HAS-BLED	65 (55.08%)	1.325 (1.015; 1.729)	0.039

Legend: AF—Atrial Fibrillation; mRS—modified Rankin Scale; HAS-BLED—Score for Hemorrhagic Risk Evaluation in Atrial Fibrillation patients.

## Data Availability

The original contributions presented in this study are included in the article. Further inquiries can be directed to Marius Militaru.
